# Complete Metabolic Remission in Metastatic BCOR-Altered Undifferentiated Round Cell Sarcoma Following Sequential Multimodal Therapy With Vincristine-Doxorubicin-Cyclophosphamide (VDC) Chemotherapy and Pazopanib Maintenance: A Case Report

**DOI:** 10.7759/cureus.107430

**Published:** 2026-04-20

**Authors:** Yatrib Chaudhary, Vidit Kapoor, Gaurav Khanna, Mannat Anand, Yashasvi Goyal

**Affiliations:** 1 Department of Oncology, Amrita Hospital, Faridabad, Faridabad, IND; 2 Department of Pathology, Amrita Hospital, Faridabad, Faridabad, IND

**Keywords:** bcor-altered sarcoma, complete metabolic response, pazopanib maintenance, undifferentiated small round cell sarcoma, vdc chemotherapy

## Abstract

BCOR-altered undifferentiated round cell sarcomas (URCS) represent a rare entity with limited epidemiological data and variable reported outcomes, contributing to the absence of standardised therapeutic strategies. We report a 27-year-old woman with metastatic BCOR-altered URCS of the scapular region who achieved a durable complete metabolic remission following a sequential multimodal treatment strategy. The patient initially received one cycle of adriamycin-ifosfamide-mesna (AIM), which was discontinued due to toxicity, followed by five cycles of vincristine-doxorubicin-cyclophosphamide (VDC), resulting in a near-complete metabolic response. This was followed by consolidation with intensity-modulated radiotherapy (52 Gy in 26 fractions). Maintenance therapy with a reduced dose of oral pazopanib 400 mg daily, based on prior reports and tolerability considerations, was administered for 12 months. Serial fluorodeoxyglucose positron emission tomography/computed tomography (FDG-PET/CT) imaging confirmed a sustained complete metabolic remission beyond 18 months with minimal toxicity. This case highlights the marked chemosensitivity of BCOR-altered URCS to anthracycline-vinca-based chemotherapy and suggests a potential role for vascular endothelial growth factor (VEGF)-directed maintenance therapy in sustaining remission.

## Introduction

Undifferentiated round cell sarcomas (URCS) comprise a heterogeneous group of high-grade mesenchymal malignancies characterised by primitive small-round-cell morphology, high nuclear to cytoplasmic ratio, and brisk mitotic activity. Their nonspecific histopathologic features and overlapping immunophenotypic profiles with other small-round-cell neoplasms, particularly Ewing sarcoma and CIC-rearranged sarcoma, often make diagnosis challenging, necessitating integration of ancillary molecular techniques for definitive classification [1,2].

Among the recently delineated molecular subsets of URCS, those harbouring BCOR (BCL6 corepressor) alterations represent a distinct clinicopathologic entity. These tumours display internal tandem duplications or fusion events such as BCOR-CCNB3 and BCOR-MAML3, which disrupt polycomb repressive complex 1.1 (PRC1.1) mediated chromatin regulation, driving global transcriptional repression of differentiation pathways [3,4]. BCOR-altered sarcomas exhibit strong nuclear BCOR expression with the absence of lineage-specific markers and frequently present with nodal or visceral metastases, reflecting a more aggressive course than Ewing-family sarcomas [5]. Optimal therapeutic strategies for BCOR-altered URCS remain undefined due to their rarity and recent molecular classification. Available studies suggest heterogeneous clinical outcomes with variable response rates and limited long-term survival data.

Current management is largely extrapolated from Ewing-family sarcoma protocols, particularly vincristine-doxorubicin-cyclophosphamide (VDC) alternating with ifosfamide-etoposide (IE) regimens, which form the cytotoxic backbone in Ewing-like tumours [6]. However, these regimens have not been rigorously assessed in BCOR-altered sarcomas, with clinical outcomes demonstrating considerable heterogeneity [7]. Nevertheless, the benefit of maintenance or anti-angiogenic therapy is uncertain, emphasising the need for detailed reports to guide evolving multimodal care. We report a rare case of metastatic BCOR-altered URCS that achieved a durable complete metabolic response following a sequential multimodal strategy. This involved a transition from an initial AIM regimen to VDC-based chemotherapy, followed by consolidative radiotherapy and maintenance pazopanib therapy. This case provides clinically informative evidence regarding treatment responsiveness and highlights the potential integration of VEGF-directed therapy in sustaining remission in this uncommon molecular entity.

## Case presentation

**Figure 1:**
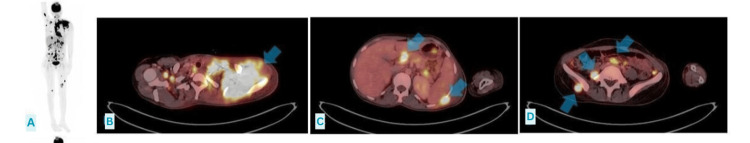
FDG PET/CT imaging demonstrating baseline disease (A-D) Baseline pre-treatment FDG PET/CT images showing extensive metabolically active disease with widespread metastases (arrows). The maximum SUVmax of the dominant lesions was 29.1. FDG PET/CT: fluorodeoxyglucose positron emission tomography/computed tomography

A 27-year-old woman with no significant past medical, surgical, or family history presented with a three-month history of progressive left shoulder pain and restricted movement. There were no constitutional symptoms such as fever, weight loss, or night sweats. On examination, her Eastern Cooperative Oncology Group (ECOG) performance status was 1. A firm, immobile, mildly tender mass was palpable over the left scapular region, with preserved overlying skin and restricted shoulder range of motion secondary to pain. No supraclavicular or axillary lymphadenopathy was detected, and systemic examination was unremarkable. A baseline whole-body fluorodeoxyglucose positron emission tomography/computed tomography (FDG-PET/CT) (Figures 1A-1D) demonstrated an FDG-avid soft-tissue mass involving (SUVmax 29.1) the left shoulder girdle, infiltrating adjacent musculature, with mixed osteolytic-sclerotic changes in the scapula and clavicle. Extensive metastatic disease was present, involving cervical, supraclavicular, infraclavicular, axillary, thoracic, mediastinal, and abdominopelvic lymph nodes, along with visceral and soft-tissue metastases in the kidneys, pancreas, peritoneum, intramuscular and subcutaneous sites, as well as left-sided pleural and pericardial effusions.

**Figure 2:**
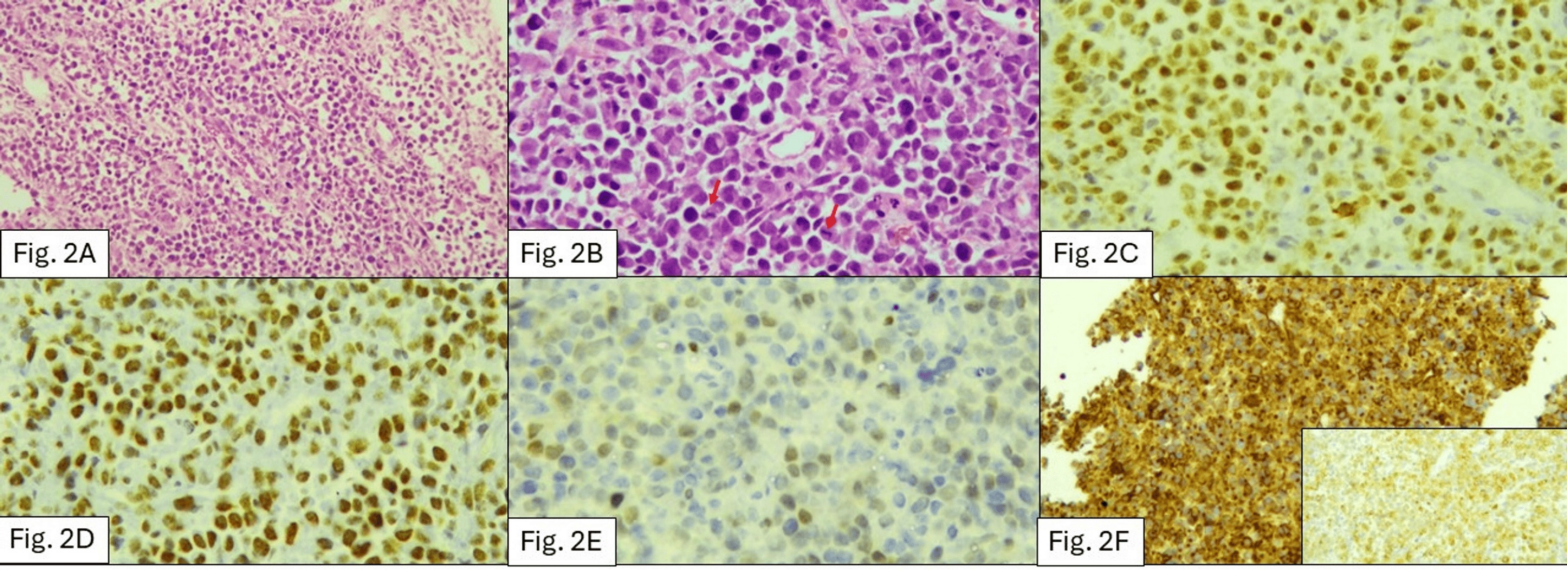
Histopathological and immunohistochemical features of the tumour (A) Hematoxylin and eosin (H&E)-stained section (×100) demonstrating a poorly cohesive population of undifferentiated tumour cells arranged in vague nesting and sheet-like patterns. (B) High-power H&E view (×400) showing tumour cells with eccentrically placed nuclei, prominent nucleoli, and rhabdoid morphology; frequent mitotic figures are noted (arrows). (C) Immunohistochemistry demonstrating strong diffuse nuclear positivity for BCOR in tumour cells. Endothelial cells within the section serve as internal negative controls. (D) INI-1 immunostaining showing retained nuclear expression in tumour cells. (E) SATB2 immunohistochemistry demonstrating weak focal nuclear positivity. (F) CD99 immunostaining showing perinuclear dot-like positivity in tumour cells. The inset demonstrates focal membranous reactivity for epithelial membrane antigen (EMA).

A core-needle biopsy of the scapular lesion revealed an undifferentiated malignant small round cell tumour composed of poorly differentiated cells in alveolar and nested patterns with rhabdoid cytomorphology, brisk mitoses, and apoptosis without necrosis. Immunohistochemistry showed strong vimentin and diffuse nuclear BCOR positivity, with heterogeneous cyclin D1, SATB2, and EMA expression. CD99 demonstrated a perinuclear dot-like pattern; NKX2.2, cytokeratin, desmin, and myogenin were negative (Figures 2A-2F), illustrating the histomorphology and immunophenotypic features. Although next-generation sequencing could not be performed owing to financial constraints, the combination of morphology and diffuse BCOR immunopositivity supported a diagnosis of BCOR-altered URCS.

**Figure 3:**
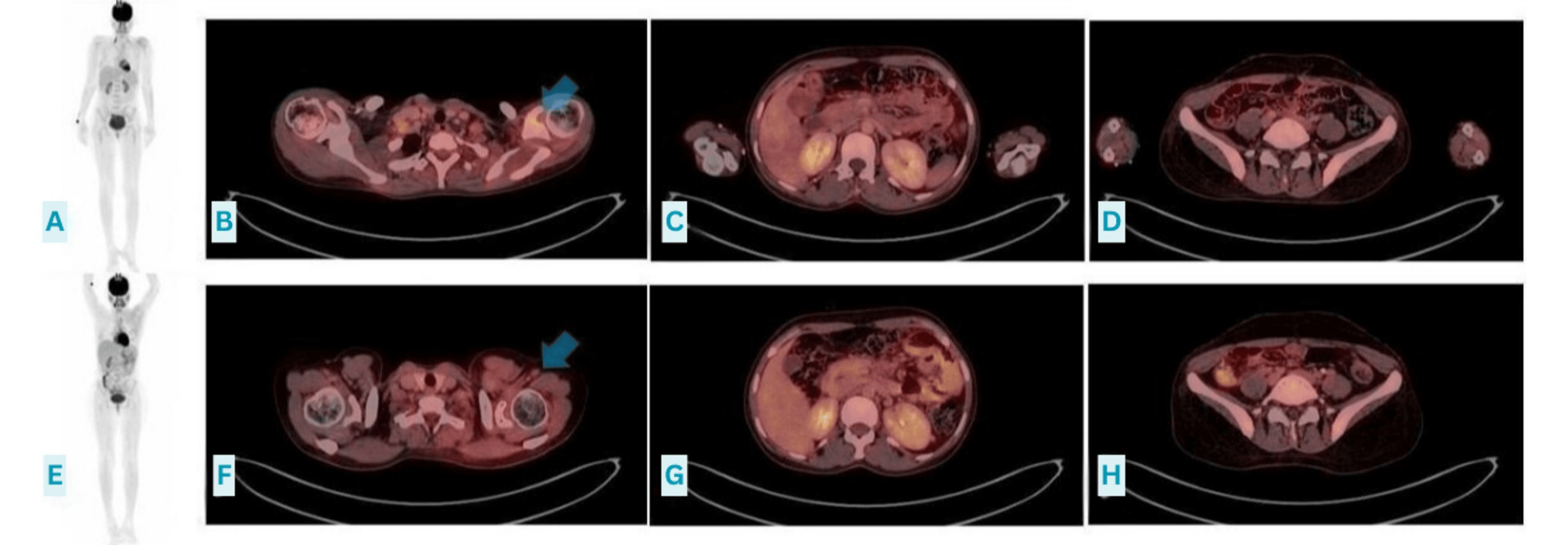
FDG PET/CT (A-D): Interim FDG PET/CT images obtained after four cycles of chemotherapy showing a very good partial metabolic response, with mild residual FDG uptake in the left shoulder region. (E-H): End-of-treatment FDG PET/CT images obtained after six cycles of chemotherapy and completion of consolidative radiotherapy to the left shoulder, demonstrating complete metabolic response with no evidence of residual hypermetabolic disease. FDG PET/CT: fluorodeoxyglucose positron emission tomography/computed tomography

Systemic therapy was initiated with the AIM regimen comprising adriamycin, ifosfamide, and mesna (AIM). The first cycle was complicated by ifosfamide-induced neurotoxicity (encephalopathy), necessitating treatment discontinuation, and associated grade III nausea/vomiting, which was resolved with prompt administration of methylene blue and supportive care. Following clinical recovery, her chemotherapy protocol was changed to VDC, of which five cycles were completed. Interim FDG PET/CT performed after four cycles of chemotherapy demonstrated a very good partial metabolic response with a reduction in SUVmax from 29.1 at baseline to ~background levels (~2-3) at interim assessment, corresponding to a >90% reduction in metabolic activity, with mild residual FDG uptake at the primary site (Figures 3A-3D). Following completion of systemic therapy, the patient underwent consolidative intensity-modulated radiotherapy (IMRT) to the left shoulder, receiving 52 Gy in 26 fractions. End-of-treatment PET/CT demonstrated a complete metabolic response with no residual FDG uptake (SUVmax approximately at background levels) with no evidence of residual hypermetabolic disease (Figures 3E-3H).

Subsequently, oral maintenance of pazopanib at 400 mg once daily was initiated and continued for 12 months, after which it was stopped. She remains on active surveillance and disease-free 18 months after completion of systemic chemotherapy and six months post completion of maintenance pazopanib.

## Discussion

The current WHO classification of soft tissue and bone tumours delineates four major molecular categories within undifferentiated small round cell sarcomas: classical Ewing sarcoma; tumours harbouring EWSR1 fusions with non-ETS partners such as NFATc2 or PATZ1; CIC-rearranged sarcomas; and sarcomas driven by BCOR genetic alterations [8]. Among these, BCOR-altered URCS constitute a distinct molecular subgroup characterised by aggressive clinical behaviour and unique molecular underpinnings [9]. Accurate diagnosis is challenging because of its morphologic and immunophenotypic overlap with Ewing sarcoma and other Ewing-like tumours. Although next-generation sequencing and fusion assays constitute the diagnostic gold standard, these methods are not universally accessible. In such settings, diffuse nuclear BCOR immunopositivity, together with the absence of lineage-specific markers such as desmin, myogenin, and cytokeratin, can serve as a practical surrogate for molecular confirmation in resource-limited settings [1,2].

In our case, the combination of strong nuclear BCOR expression and characteristic histopathologic features enabled a confident diagnosis despite the absence of molecular testing. Similar reliance on BCOR immunohistochemistry has been reported in recent series, underscoring its reproducibility and specificity for this subset [3,5]. However, given the known morphologic and immunophenotypic overlap, careful consideration of differential diagnoses such as Ewing sarcoma and CIC-rearranged sarcoma is essential to avoid potential diagnostic pitfalls. This resource-adapted approach highlights the need to integrate morphology with surrogate molecular markers for accurate subclassification of rare sarcomas in real-world practice.

In current clinical practice, Ewing sarcoma-based multi-agent chemotherapy protocols are most frequently employed for systemic therapy in BCOR::CCNB3 sarcoma [10]. Combination chemotherapy with doxorubicin and ifosfamide, most commonly administered as the AIM regimen, remains a standard first-line option for advanced or high-grade soft-tissue sarcomas [11,12]. However, its true efficacy in BCOR-altered URCS is uncertain, as most available data derive from morphologically rather than molecularly defined sarcoma cohorts [2]. Furthermore, emerging molecular analyses suggest that BCOR-altered sarcomas harbour distinctive transcriptional repression programs and altered DNA-repair pathways, which may reduce sensitivity to ifosfamide-dominant regimens [5].

In our patient, AIM therapy was discontinued after two days because of ifosfamide-related neurotoxicity, precluding meaningful assessment of treatment response. A study established VDC as the cytotoxic backbone of Ewing-family sarcoma treatment, prompting the early transition to this regimen in our patient [6]. Following VDC, restaging FDG PET/CT demonstrated a near-complete metabolic remission (CMR), confirming marked chemosensitivity to anthracycline-vinca-based therapy. Responses to VDC- or VDC/IE-based regimens have been reported in BCOR-CCNB3 and BCOR-ITD sarcomas [3,13]. Taken together, these observations suggest that BCOR-altered URCS may exhibit clinically meaningful sensitivity to anthracycline-vinca-containing combinations, underscoring the importance of early, response-guided treatment adaptation.

Local radiotherapy is a key component of multimodal management in URCS, consolidating systemic responses and securing durable local control. In our patient, IMRT to the scapular primary was delivered after completion of chemotherapy. Although metastatic disease is frequently approached with palliative intent, data from Ewing-family and soft-tissue sarcoma cohorts indicate that definitive local control can prolong progression-free survival when systemic remission has been achieved [6,14]. In this setting, radiotherapy not only reduces the risk of local relapse but also reinforces systemic control by helping maintain CMR. The use of IMRT in our case, therefore, represents a critical link between chemotherapy-induced remission and durable local disease control. The decision to introduce pazopanib as maintenance therapy was informed by the central role of angiogenesis in sarcoma biology and emerging evidence of VEGF-pathway dysregulation in BCOR-altered tumours. BCOR-altered disrupts PRC1.1-mediated chromatin regulation, generating aberrant transcriptional programs that include upregulation of pro-angiogenic pathways [4]. This biological context supports targeting the vascular endothelial growth factor (VEGF) axis to suppress micrometastatic disease and sustain remission after cytotoxic therapy. The EORTC 62072 (PALETTE) trial established pazopanib, a multitargeted tyrosine-kinase inhibitor of VEGFR-1/2/3, PDGFR, and c-KIT, as an effective second-line agent in advanced soft-tissue sarcomas, demonstrating a significant improvement in progression-free survival versus placebo [15]. Subsequent real-world series and maintenance-phase analyses have further supported its favourable tolerability and disease-stabilising potential in patients who achieve good responses to prior chemotherapy [16].

In our patient, sequential VDC-based chemotherapy followed by consolidative IMRT and maintenance pazopanib achieved a durable CMR sustained for more than 18 months, with excellent tolerability. This clinical course suggests that BCOR-altered sarcomas may retain angiogenic dependence even after systemic remission and that VEGF inhibition may serve as a rational bridge between cytotoxic therapy and prolonged disease quiescence. Clinically, this case illustrates a biologically coherent, adaptive multimodal strategy integrating systemic cytotoxic therapy, definitive local control, and targeted maintenance therapy. It highlights guiding principles: effectiveness of anthracycline-vinca-based therapy in this disease population, response-adapted sequencing supported by metabolic imaging, and consideration of anti-angiogenic maintenance to sustain remission in chemosensitive disease.

In summary, our experience suggests that precision-aligned multimodality treatment anchored in metabolic monitoring and VEGF-directed maintenance can achieve sustained remission even in metastatic BCOR-altered URCS. These observations support further prospective and registry-based evaluation of biology-informed therapeutic strategies in this molecular sarcoma subset. While causality cannot be inferred from a single case, the sustained remission observed here supports further evaluation of maintenance anti-angiogenic strategies in molecularly defined, chemosensitive BCOR-altered sarcomas.

Our findings highlight that BCOR-altered URCS is a rare and aggressive molecular subtype with limited therapeutic evidence. Anthracycline-vinca-based chemotherapy (VDC) demonstrated significant chemosensitivity in this case. Treatment adaptation was guided by toxicity, with discontinuation of ifosfamide due to neurotoxicity. Multimodal therapy, including chemotherapy, radiotherapy, and pazopanib maintenance, resulted in durable CMR. Pazopanib maintenance may have a potential role in sustaining remission in BCOR-altered sarcomas; however, evidence remains limited.

## Conclusions

This case highlights a durable CMR in metastatic BCOR-altered URCS achieved through a multimodal sequence of VDC chemotherapy, consolidative IMRT, and pazopanib maintenance. The sustained remission and favourable tolerability suggest that BCOR-altered sarcomas can exhibit meaningful chemosensitivity. While conclusions from a single case are inherently exploratory, our findings suggest a potential role for VEGF-pathway inhibition and warrant further prospective evaluation in this sarcoma subtype.
